# The Efficacy of Acceptance and Commitment Therapy for Transitional-Age Youth: A Meta-analysis

**DOI:** 10.1007/s10567-025-00543-5

**Published:** 2025-09-03

**Authors:** Janna Keulen, Maja Deković, Matthijs Oud, Jacqueline A-Tjak, Denise Bodden

**Affiliations:** 1 Department of Clinical Child & Family Studies, Utrecht University, Utrecht, The Netherlands; 2 Department of Care and Participation, Trimbos Institute, Utrecht, The Netherlands; 3 A-Tjak Cursussen, Zeist, The Netherlands

**Keywords:** Acceptance and commitment therapy, Transitional-age youth, Adolescents, Young adults, Meta-analysis

## Abstract

This meta-analysis integrated the findings on the efficacy of acceptance and commitment therapy (ACT) for transitional-age youth (TAY; youth aged 15 to 25) on psychopathology (i.e., internalizing, externalizing and other psychological problems), ACT related processes (i.e., psychological flexibility and self-compassion), well-being (i.e., general and social well-being) and coping (i.e., emotional and cognitive coping). Additionally, we used meta-regression analyses to examine whether effect sizes varied based on the type of (sub) outcome, timing of assessment, various intervention characteristics, type of control group and several sample characteristics. We executed a three-level meta-analytic model in R. Based on 65 studies (*n* = 5283), we found a moderate effect (Hedges’s g = 0.72) of ACT compared to the control conditions on psychopathology, ACT related processes, well-being and coping. The quality of the evidence was very low due to a relatively high risk of bias in the selected studies, considerable heterogeneity in effect sizes and a risk of publication bias. Regarding the meta-regression analyses, we found that ACT was more effective than waitlist and TAU conditions, but equally effective compared to CBT, other treatments (e.g., Rational Emotive Behavior Therapy) and other control conditions (e.g., educational intervention). Our results suggest that ACT is an effective intervention for reducing psychopathology and increasing ACT related processes, well-being and coping in TAY with diverse types and severity of psychological problems. We recommend future research to conduct more high quality research, including larges samples, active control conditions, longer follow-up periods and measures of treatment integrity, in more diverse populations of TAY.

Acceptance and Commitment Therapy (ACT) is a third-wave cognitive behavioral therapy (CBT) developed in the late twentieth century (Hayes et al., 1999, 2004). Unlike traditional therapies focusing on symptom reduction, ACT aims to enhance psychological flexibility: “the ability to contact the present moment more fully as a conscious human being, and to change or persist in behavior when doing so serves valued ends” (Hayes et al., 2006, p. 7). This is achieved through six core processes: acceptance (i.e., accepting unpleasant emotions, thoughts, and situations), defusion (i.e., altering the unwanted functions of thoughts), present moment awareness (i.e., focusing on the here-and-now), self-as-context (i.e., cultivating a flexible perspective of the self, where self-related content can be observed and accepted), values (i.e., identifying what is most important for oneself), and committed action (i.e., adjusting behavior in alignment with one’s values). ACT’s transdiagnostic approach makes it applicable across diverse psychological and physical conditions, including pain, depression, and stress (Gloster et al., 2020).

Although ACT is a relatively new form of CBT, the interest in ACT has grown in the past years and more and more meta-analyses have been conducted on the efficacy of ACT (e.g., A-Tjak et al., 2015; Öst, 2008, 2014; Powers et al., 2009). In a review of meta-analyses, Gloster et al. (2020) concluded that ACT is equally effective as established evidence-based treatments (e.g., CBT) and superior to inactive control conditions (e.g., placebo or waitlist) and treatment as usual (TAU). Moreover, recently, Levin et al. (2024) provided an overview on ACT research and concluded that ACT (including alternative, scalable formats like digital self-help, as well as its delivery by non-mental health professionals) can be used to effectively treat a wide range of psychological problems.

Most of the aforementioned studies, however, have been conducted with adult samples. There are fewer meta-analyses investigating the efficacy of ACT in younger populations (i.e., adolescent and young adults; Fang & Ding, 2020; Howell & Passmore, 2019; Parmar et al., 2021; Perkins et al., 2022; Wang & Fang, 2023). The authors of these studies concluded that, although more high-quality research is needed, ACT also appears to be an effective intervention for adolescents and young adults. Nevertheless, the results of these earlier meta-analyses are limited in their generalizability. Particularly, they either focused on specific target populations (e.g., children with special needs [Parmar et al., 2021] or university students [Howell & Passmore, 2019]), particular delivery formats (e.g. internet-based ACT [Wang & Fang, 2023]) or studied third-wave CBT in general (Perkins et al., 2022). Furthermore, some of them conducted their literature searches more than five years ago (Fang & Ding, 2020; Howell & Passmore, 2019), missing many recent studies as the research on the efficacy of ACT is rapidly growing. In addition, the meta-analyses that specifically focused on ACT (i.e., as opposed to third wave CBT in general) all included less than 15 studies and had therefore limited capacity for conducting meta-regression analyses (i.e., only meta-regression analyses regarding study quality, type of control condition and type of target group were performed). Hence, conducting a new meta-analysis that specifically focuses on ACT, while incorporating a wider range of youth populations (e.g., clinical and non-clinical samples), different delivery formats (e.g., online and offline ACT) and more extensive meta-regression analyses (e.g., various intervention and sample characteristics), seems warranted.

Moreover, these earlier meta-analyses focused either on adolescents below the age of 18 or on young adults above the age of 18. To our knowledge, there are no meta-analyses performed in transitional-age youth (TAY; youth aged 15 to 25; Wilens & Rosenbaum, 2013), while studying the effects of ACT in this specific age group is especially relevant. TAY represent an age group with unique developmental tasks, including identity formation and the pursuit for independence. Moreover, as the transitional age period involves significant psychological and social changes, TAY are more vulnerable to develop psychological problems compared to other age groups (Whiteford et al., 2013; Wilens & Rosenbaum, 2013). This highlights the importance of studying the effects of interventions that support TAY in their transition to adulthood.

Hence, the first goal of the current meta-analysis was to integrate the findings on the efficacy of ACT for TAY on psychopathology (i.e., internalizing, externalizing and other psychological problems), ACT related processes (i.e., psychological flexibility and self-compassion), well-being (i.e., general and social well-being) and coping (i.e., emotional and cognitive coping). Based on earlier research, we expected that for TAY, ACT is more effective than waitlist and TAU conditions and equally effective compared to well established evidence based treatments (e.g., CBT). The second goal of the meta-analysis was to explain potential heterogeneity in effect sizes by conducting various meta-regression analyses. To our knowledge, most meta-analyses that performed meta-regression analyses have focused exclusively on studies in adult populations. By performing meta-regression analyses, we can better understand the results of the studies and detect for whom and under what circumstance ACT is most effective for TAY (Kraemer et al., 2002).

First, we examined whether the effect sizes were different for our main and sub outcomes: psychopathology (sub outcomes are internalizing, externalizing and other psychological problems), ACT related processes (sub outcomes are psychological flexibility and self-compassion), well-being (sub outcomes are general and social well-being) and coping (sub outcomes are emotional and cognitive coping). Some meta-analyses found that the effects of ACT were different for various types of psychological problems. For instance, Powers et al. (2009) found ACT to be effective for depression, physical health and other mental health problems but not for distress problems. In addition, Öst (2014) found ACT to be more effective for psychiatric disorders than for somatic disorders and work-related stress. However, to our knowledge, there are no studies yet comparing the effects of ACT on our specified main and sub outcomes. Hence, the meta-regression analyses regarding the type of main outcome, type of psychopathology, type of ACT related process, type of well-being and type of coping, in this specific age group, were conducted in an exploratory manner.

Second, we investigated whether effect sizes varied depending on the timing of assessment (i.e., post-intervention, one-week to one-month follow-ups, one- to three-month follow-ups, and three- to twelve-month follow-ups). We anticipated no differences in effect sizes between post-intervention and follow-up assessments (A-Tjak et al., 2015; Powers et al., 2009).

Third, we studied whether effect sizes differed across various intervention characteristics, including guidance (guided or unguided ACT), delivery format (offline, online, or blended ACT), therapy format (group or individual ACT), duration of the intervention, the number of targeted psychological flexibility processes and treatment integrity. Based on earlier meta-analyses, we expected that studies investigating the efficacy of interventions with therapist guidance (French et al., 2017; Spijkerman et al., 2016; Thompson et al., 2021) and higher levels of treatment integrity (Power et al., 2022) show larger effect sizes compared to studies examining the effects of interventions without therapist guidance (e.g., self-help books or apps) and with lower levels of treatment integrity. We did not expect different effect sizes regarding delivery format (Di Sante et al., 2022; French et al., 2017; Han & Kim, 2022) and therapy format (Öst, 2014; Perkins et al., 2022; Ruiz, 2012). Additionally, due to no or mixed findings in earlier research, the meta-regression analyses of intervention duration and the number of targeted psychological flexibility processes were conducted in an exploratory manner. While some meta-analyses suggest that intervention duration is positively related to efficacy (Di Sante et al., 2022; Prudenzi et al., 2021; Spijkerman et al., 2016), others did not find this relationship (Öst, 2014; Ruiz, 2012).

Fourth, we examined whether effect sizes differed when ACT was compared to various types of control groups (i.e., waitlist, TAU, CBT, other treatment or other control group). Consistent with previous meta-analyses on ACT, we expected the largest effect sizes for studies comparing ACT with a waitlist condition, smaller effects sizes for studies comparing ACT with TAU, and the smallest effect sizes for studies comparing ACT with established treatments (i.e., CBT and other treatment; e.g., Fang & Ding, 2020; Gloster et al., 2020).

Last, we conducted several meta-regression analyses to examine potential differences in effect sizes across various sample characteristics, including mean age, gender, target group (non-clinical, sub-clinical, clinical sample or mixed) and presence of comorbidity. Due to contrasting or insufficient evidence in earlier research, these meta-regression analyses were conducted in an exploratory manner. Specifically, although some prior meta-analyses suggest ACT’s efficacy varies by age, favoring adults over youth (Bai et al., 2020; Dawson et al., 2022), other meta-analyses have not found such differences (Öst, 2014; Perkins et al., 2022; Ruiz, 2012). Moreover, while Öst (2014) found higher effect sizes in studies with higher proportions of females, Ruiz (2012) did not find any differences related to gender. Additionally, where some meta-analyses concluded that ACT may be more effective for patients with mild to moderate depressive symptoms than for patients with moderate to severe depressive symptoms (Bai et al., 2020; Sun et al., 2022), other meta-analyses found no differences related to target group (Dawson et al., 2022; Öst, 2014; Perkins et al., 2022) or even larger effect sizes for studies targeting clinical samples than for studies targeting non-clinical samples (Han & Kim, 2022). Last, to our knowledge, no meta-analyses have yet conducted a meta-regression analysis on comorbidity.

## Method

This meta-analysis was reported in line with the Preferred Reporting Items for Systematic Reviews and Meta-analyses (PRISMA) guidelines (Page et al., 2021). The meta-analysis was pre-registered in PROSPERO (registration number CRD42022304276).

## Search Strategy

The following databases were electronically searched for published literature: PsycINFO, Pubmed, Web of Science and Scopus. The final search was conducted in November 2023. The search string can be found in Appendix A. After the screening (January 2024) we also searched for relevant articles on the webpage of the Association for Contextual Behaviour Science (ACBS, 2023). This website contains a list of all RCTs investigating the effectiveness of ACT and its components that have been conducted since 1986.

## Study Selection

Studies were selected for inclusion if they (1) were written in English, (2) had a mean age between 14.5 and 25.5 years old, (3) investigated the efficacy of ACT (i.e., studies investigating the efficacy of other third wave therapies, such as acceptance-based behavior therapy, were excluded), (4) were (cluster-)randomized controlled trials, (5) investigated ACT independently and not in combination with another therapy (except for stable medication), (6) randomized at least 10 participants to each condition and (7) reported sufficient quantitative information about one of our outcomes. We did not include grey literature or searched for unpublished data. Additionally, we adjusted the mean age inclusion criterion from 15 to 25 years (see pre-registration) to 14.5 to 25.5 years, allowing us to include nine more studies and thereby increasing the power of our meta-regression analyses. The first author initially screened all studies based on their titles and abstracts and then screened the remaining studies based on their full-texts. To calculate interrater reliability, two additional independent researchers screened 27.3% of the 4022 titles and abstracts and 25.6% of the 868 full-texts. If the full-text or relevant information (e.g., the mean age of the sample) was missing, we contacted the authors. The studies of authors who did not respond before April 2024 were not included in the meta-analysis.

## Data Extraction

The first author coded all studies using a detailed coding sheet (this coding sheet can be requested by emailing the corresponding author). To calculate interrater reliability, the last author also coded the effect sizes and variances for all type of (sub) outcomes, timing of assessments, intervention characteristics, type of control groups and sample characteristics of 24.6% of the 65 included studies. Moreover, another independent researcher additionally coded the risk of bias of 27.7% of the 65 included studies.

### Outcomes

To calculate the effect sizes and variances of our outcomes, we extracted the means, standard deviations and number of participants at pretest, posttest, and follow-up(s) for both the ACT and control groups from the article. When a standard error was reported instead of a standard deviation, we calculated the standard deviation using the formula: $SD=SE*\surd N$. If the means, standard deviations, and/or number of participants were not clearly reported in the article, we contacted the authors. Effect sizes from studies whose authors did not respond by April 2024 were excluded from the meta-analysis.

In general, all outcomes were analyzed together and an overall effect of ACT was calculated, but meta-regression analyses were performed to investigate if effect sizes differed between our main and sub outcomes. Specifically, we divided the study outcomes into four main outcomes and nine sub outcomes. To categorize the (sub) outcomes, the first, second and last author independently categorized all study outcomes in separate outcome clusters. Based on the results of this clustering and through discussion, the final four categories and nine subcategories were composed. “Type of outcome” was categorized as psychopathology, ACT related processes, well-being and coping. “Type of psychopathology” was labeled as internalizing (e.g., depression), externalizing (e.g., aggression) or other (e.g., hoarding). Moreover, “type of ACT related process” was coded as psychological flexibility (e.g., acceptance) or self-compassion. We categorized “type of well-being” as general (e.g., happiness) or social (e.g., social functioning) well-being and “type of coping” as emotional (e.g., emotion regulation) and cognitive (e.g., repetitive negative thinking) coping. In Appendix B, we presented the distribution of all study outcomes across the various (sub) outcome clusters.

### Timing of Assessment

“Timing of the outcome assessment” was coded as post-intervention, one week to one month follow-up, one to three months follow-up or three to twelve months follow-up.

### Intervention Characteristics

“Guidance” was categorized as guided (i.e., guided interventions or self-help interventions with additional guidance) or unguided (i.e., clients did not receive any guidance during the ACT intervention, such as self-help books, apps or websites). We coded “delivery format” as offline, online, or blended (i.e., both offline as online) and “therapy format” as group or individual therapy. “Duration of the treatment” and “number of psychological flexibility processes” were coded as continuous variables. The duration of the treatment was calculated by multiplying the duration of one session (in hours) with the total number of sessions. The number of psychological flexibility processes was coded by checking how many of the six core principles of psychological flexibility (i.e., acceptance, defusion, self-as-context, present moment, values, committed action) were included in the intervention. For “treatment integrity”, we initially documented the authors’ descriptions on this aspect and later explored how this could be integrated into one variable.

### Type of Control Group

“Type of control group” was coded as belonging to one of the following categories: waitlist, TAU, CBT, other treatment (e.g., mindfulness, rational emotive behavior therapy [REBT]) or other control group (e.g., educational intervention, reflection seminars).

### Sample Characteristics

The mean age of the participants at the start of the study was coded as a continuous variable. Gender was coded as the percentage of females in the sample. “Target group” was categorized as a non-clinical (i.e., participants are not screened on psychological problems; everyone can join the study), sub-clinical (i.e., participants have at least subclinical scores on a screening measure), clinical (i.e., participants receive treatment at a mental healthcare institution or have clinical scores on a screening measure) or mixed. We coded “presence of comorbidity” as yes (i.e., participants had more than one diagnosis) or no (i.e., participants had only one diagnosis).

### Risk of Bias

Risk of bias was coded with version one of the Cochrane risk-of-bias tool for randomized trials (Higgins et al., 2011). Each study was rated on the following six domains: random sequence generation, allocation concealment, blinding of participants and personnel, blinding of outcome assessment, incomplete outcome data (this was assessed as low risk when intention-to-treat analyses were conducted) and selective outcome reporting. The last domain of the risk-of-bias tool concerning other potential biases was omitted in this study as we found no explicit indication in any of the studies suggesting that this factor had affected the study’s validity.

### Interrater Reliability

For reliable study selection and data extraction, we evaluated the interrater reliability between the raters. For the full-text screening, the inter-rater reliability was corrected for prevalence bias due to the high rate of exclusion over inclusion (Hallgren, 2012). There was a substantial inter-rater reliability for both the title and abstract screening (based on 27.3% of abstracts: 85.5% agreement, κ = 0.72) as the full-text screening (based on 25.6% of full-texts: 87.8% agreement, prevalence corrected κ = 0.76; Landis & Koch, 1977). Regarding the data extraction, the ICCs were calculated using a two-way random-effect model. The interrater reliability for the continuous variables was excellent (based on 24.6% of the studies): hedges’ g (ICC = .93), sampling variance (ICC = .98), number of psychological flexibility processes (ICC = .95), duration (ICC = 1.00), mean age (ICC = .98) and gender (ICC = 1.00). The interrater reliability for the categorical variables were substantial to perfect (based on 24.6% of the studies): type of outcome (κ = .97), type of psychopathology (κ = .63), type of ACT related process (κ = 1.0), type of well-being (κ = 1.0), type of coping (κ = 1.0), timing of assessment (κ = 1.0), guidance (κ = .72), delivery format (κ = 1.0), therapy format (κ = .87), type of control group (κ = 1.0) and target group (κ = .91). The interrater reliability for the risk of bias domains were moderate to perfect (based on 27.7% of the studies): random sequence generation (κ = 1.0), allocation concealment (κ = .81), blinding participants (κ = 1.0), blinding outcome assessment (κ = 1.0), incomplete outcome data at posttest (κ = .61), incomplete outcome data at first follow up (κ = .60) and selective outcome reporting (κ = .44). In all steps, discrepancies were resolved through discussion among the raters.

### Quality of Evidence

The quality of evidence of the overall effect of ACT on all outcomes was rated by the first and third author using the Grades of Recommendation, Assessment, Development and Evaluation (GRADE) approach (Guyatt et al., 2011). The GRADE approach is a structured assessment of quality of evidence and has four levels of evidence: very low, low, moderate and high. In GRADE, evidence from randomized controlled trials starts at high evidence and can be downgraded in the case of risk of bias, inconsistency of finding, indirectness, imprecision and publication bias.

## Data Analysis

### Effect Size Calculation

First, cohen’s *d* effect sizes were calculated using the formula of Lipsey and Wilson (2001): $d=\frac{Mact-Mcontrol}{SDpooled}$. Following the approach suggested by Morris (2008), we calculated the effect size by subtracting the mean pre-to-post or pre-to-follow-up change in the control group from that in the ACT group, divided by the pooled pretest standard deviation. Second, to control for small sample sizes, all effect sizes were adjusted using Hedges (1983) small sample size correction using the formula of Lipsey and Wilson (2001): $Hedge{s}{\prime}g=d*\left(1-\left(\frac{3}{4\left(n act+n control-2\right)-1}\right)\right)$. According to the criteria formulated by Cohen (1988), Hedges’ g = .2, Hedges’ g = .5, and Hedges’ g = .8 were interpreted as small, moderate, and large effects respectively. If a study included two or more outcomes or time assessments, separate effect sizes were calculated. Positive effect sizes represented more beneficial results for the ACT condition compared to the control condition(s).

### Three-Level Meta-Analysis

To account for dependency in the data and be able to include multiple effect sizes per study, we executed a three-level meta-analytic model in R (Assink & Wibbelink, 2016; Van den Noortgate et al., 2013). In this model, the first level represented the sampling variance of each effect size, the second level characterized the within-study variance of effects sizes within the same study and the third level represented the between-study variance of effects sizes from different studies. The overall effect size was calculated by using the intercept only model. To investigate if there was significant heterogeneity within (i.e., level 2) and/or between (i.e., level 3) studies, we executed separate log-likelihood tests. If there was evidence for heterogeneity in effect sizes at one of the two levels, meta-regression analyses were conducted. For categorical predictors, we computed dummy variables and continuous predictors were centered around their mean. Meta-regression analyses were only performed in the three-level intercept model if there were at least three effect sizes for each level of the specific predictor (Crocetti, 2016). Last, Hox (2010) suggests that in meta-analyses, intertwined predictors could often lead to multicollinearity issues, making it challenging to pinpoint relevant effects. Hence, after evaluating all predictors in separate models, we tested all significant predictors in one single model to determine whether results remained significant when controlling for the other significant predictors. If a predictor is no longer significant in the meta-regression that includes all significant predictors, it suggests that this predictor may not be the most important factor in explaining the heterogeneity in effect sizes, and that this heterogeneity may be better explained by the other predictors in the model. To test if multicollinearity was not too high, and results of the multiple predictor model were still reliable, we calculated Variation Inflation Factors (VIF). VIF values ranging from 1 to 5 indicate moderate correlations between predictors, while values above 5 suggest high correlations among predictors (Kutner et al., 2004).

### Publication Bias

Regarding publication bias, we visually inspected the funnel plot where effect sizes were plotted against their precision (standard errors; Light & Pillemer, 1984). Second, the Egger’s asymmetry test was performed to statistically test if the funnel plot was asymmetrical (Egger et al., 1997). In multilevel meta-analyses, the assumption of independence among effect sizes challenges traditional tests for publication bias. To address this, in line with other multilevel meta-analyses (e.g., De Jong et al., 2021; Ostrowski et al., 2022; Wilkop et al., 2023), we followed the suggestion of Viechtbauer (2015) and included the sampling variance of the effect sizes as a moderator in the random-effects model. A significant deviation of the intercept from zero would suggest asymmetry. Third, if Egger’s regression test was significant, a trim-and-fill analysis was performed to adjust the effects for possible publication bias (Duval & Tweedie, 2000a, 2000b). Previous studies have shown that effect size estimates based on imputation after this procedure may not be accurate for multilevel meta-analyses (Fernández-Castilla et al., 2021). Therefore, we followed the method by Fernández-Castilla et al. (2021), which imputes effect sizes at either the left or right tail of the distribution.

## Results

**Fig. 1:**
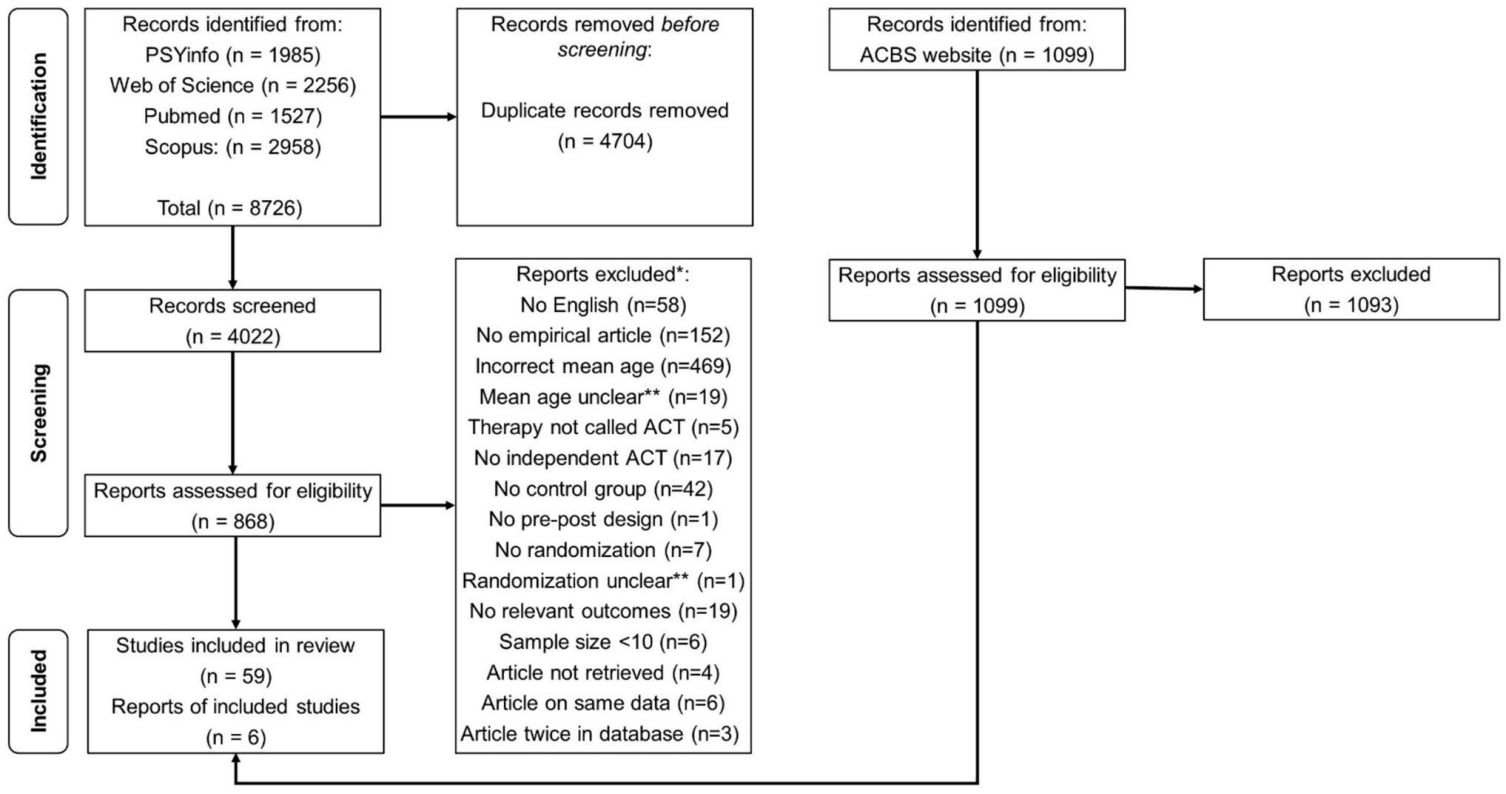
PRISMA Flowchart of the Screening Process. *Articles were screened in the following order on eligibility: language, type of article, mean age, name of the intervention, independence, control group, pre-post design, randomization, relevant outcomes, sample size at pre- and post-intervention assessment. **Authors were emailed for relevant information and reminded twice. If they did not respond before April 2024, the article was not included

A total of 4022 records were screened based on their titles and abstracts, and 868 studies were assessed for eligibility based on their full-texts. Ultimately, 59 studies were selected through the search string, with an additional 6 studies retrieved from the ACBS website (ACBS, 2013). Figure 1 provides an overview of the selection process and reasons for exclusion.

## Characteristics of the Selected Studies

Table 1 shows the characteristics of the studies that are included in the meta-analysis. There were 65 studies, with two studies investigating the effects of ACT in two independent samples. Hence, in total there were 67 samples and 584 effect sizes, representing 5283 individuals. Specifically, the effects of ACT on psychopathology were examined in 56 studies (*n* = 4768), on ACT-related processes in 42 studies (*n* = 3745), on well-being in 31 studies (*n* = 2350) and on coping in 15 studies (*n* = 1036). On average, the mean age of participants in the studies was 20.36 (based on 64 samples) with 72.7% of the participants being female (based on 65 samples). In addition, 19.7% of the participants was part of an ethnic minority within the country the study was conducted (based on 25 samples) and within most samples, the majority of participants attended university, college or high-school (i.e., 26 samples mainly included university students/graduates, 12 samples mainly included college students/graduates, 17 samples mainly included high school students). The publication year of the included studies ranged from 2011 to 2024. Almost all studies randomized participants on an individual level. Only six studies used group randomization. In total, 21 studies were conducted in Anglosphere countries (e.g., Australia, United States, Canada and England), 15 studies in West European countries (e.g., The Netherlands, Belgium, Sweden, Switzerland), 2 studies in East European countries (i.e., Cyprus), 2 studies in Latin America (i.e., Colombia), 2 studies in Sub-Saharan Africa (i.e., Nigeria and Ethiopia), 4 studies in the Middle East (i.e., Turkey and Egypt), 17 studies in Southern Asia (e.g., Iran and Malaysia) and 2 studies in Confucian Asia (i.e., China and Japan).

**Table 1:** Characteristics of the Included Studies Used in the Meta-Analysis

Authors (year)	N ES	N ACT	N control	Mean age (*SD*)	Age range	% Female	Control group(s)	Sub outcomes	Guidance	Delivery format	Therapy format	Duration in hours	Timing measurements
Asale et al. (2021)	7	29	20	16.8 (1.11)	–	100	Waitlist	Psychological flexibility General well-being Social well-being Emotional coping	Guided	Offline	Group	20.0	Post
Van Aubel et al. (2020)	16	27	28	21.0 (2.43)	16;25	72.7	Other (structured group discussion about documentaries)	Internalizing problems Other psychological problems Psychological flexibility	Guided	Blended	Group	7.5	Post 3 months to one year FU
Azadeh et al. (2016)	2	15	15	15.4 (0.78)	15;16	100	Waitlist	Psychological flexibility Social well-being	Guided	Offline	Group	15.0	Post
Barreto and Gaynor (2023)	5	22	23	22.4 (6.86)	–;–	77.3	Other (informational handout with tips on how to improve their health)	Other psychological problems Psychological flexibility General well-being	Guided	Offline	Individual	1.0	Post 1 week to 1 month FU
Bernal-Manrique et al. (2020)	6	21	21	14.5 (1.67)	11;17	71.4	Waitlist	Internalizing problems Psychological flexibility Emotional coping Cognitive coping	Guided	Offline	Group	3.8	Post
Butryn et al. (2011)	6	35	19	23.1 (3.80)	–;–	100	Other (education intervention about safely engaging in physical activity)	Psychological flexibility	Guided	Offline	Group	4.0	Post 1 week to 1 month FU
Değerli and Odacı (2024)	9	16	16	20.4 (0.61)	–;–	75.0	Waitlist	Psychological flexibility Self-compassion Social well-being	Guided	Offline	Group	16.7	Post 1 to 3 month FU
Dereix-Calonge et al. (2019)	5	43	42	23.4 (2.87)	20;33	79.0	Waitlist	Internalizing problems Psychological flexibility Cognitive coping	Guided	Offline	Group	6.0	Post
Ditton et al. (2023)	18	37;36	35	24.0 (5.48)	18;46	68.1	Waitlist	Internalizing problems Psychological flexibility General well-being	Unguided	Online	Individual	–	Post
Ekeanya et al. (2023)	2	23	26;22	–	15;19	57.1	Other treatment (rational emotive behaviour therapy) Other (educational talks and improvement of academic performance)	General well-being	–	–	–	–	Post
El-Ashry et al. (2023)	4	42	39	22.1 (1.34)	–	71.4	Waitlist	Psychological flexibility Social well-being	Guided	Online	Group	7.5	Post 1 week to 1 month FU
Enayati et al. (2018)	4	30	30	16.4 (0.591)	–	100	Waitlist	Psychological flexibility General well-being	Guided	Offline	–	16.0	Post Unknown FU
Engström et al. (2022)	8	52	47	24.4 (4.4)	–;–	100	TAU	Internalizing problems Other psychological problems Psychological flexibility	Guided	Online	Individual	21.0	Post 3 months to 1 year FU
Fabricant et al. (2013)	2	40	27;9	20.7 (–)	18;53	39.3	Other treatment (exposure) Other (expressive writing)	Internalizing problems Cognitive coping	Guided	Offline	Individual	0.8	Post
Fang et al. (2023)	14	48	48;48	19.7 (1.46)		51.1	Other treatment (rational emotive behavior therapy) Waitlist	Internalizing problems Other psychological problems Psychological flexibility Emotional coping	Guided	Offline	Group	12.0	Post
Frögéli et al. (2016)	8	69	44	24.7 (6.90)	18;46	–	Other (reflection seminars for personal and professional development)	Internalizing problems Psychological flexibility	Guided	Offline	Group	12.0	Post 1 to 3 month FU
Ghasemi et al. (2023)	8	15	15;15	16.1 (3.21)	–	100	Other treatment (mindfulness) Waitlist	Internalizing problems Cognitive coping	Guided	Online	Group	–	Post Unknown FU
Gloster et al. (2017)	8	21	23	22.3 (–)	18;40	71.4	Waitlist	Internalizing problems Psychological flexibility	Guided	Offline	Group	12.0	Post 1 week to 1 month FU
Van der Gucht et al. (2017)	22	288	298	17.0 (0.66)	14;21	53	Waitlist	Internalizing problems Externalizing problems Other psychological problems Psychological flexibility General well-being Social well-being	Guided	Offline	Group	8.0	Post 3 months to one year FU
Hashemipoor et al. (2021)	8	15	15	22.0 (–)	18;35	48.0	Unclear	Other psychological problems General well-being Social well-being	Guided	Offline	Group	–	Post 1 to 3 month FU
Hayes et al. (2011)	4	22	16	14.9 (2.55)	12;18	55.3	TAU	Internalizing problems Other psychological problems	Guided	Offline	Individual	9.3	Post 1 to 3 month FU
Ito and Muto (2020)	16	14	12	19.9 (1.23)	–	61.5	Waitlist	Internalizing problems Psychological flexibility General well-being Social well-being	Guided	Offline	Individual	–	Post 1 to 3 month FU
Karaaziz et al. (2023)	4	24	21	24.3 (–)	–;–	59.0	Waitlist	Internalizing problems Psychological flexibility	Guided	Offline	Group	3.0	Post 1 week to 1 month FU
Karekla et al. (2022)	6	62	30	15.3 (2.15)	–;–	100	Waitlist	Internalizing problems Psychological flexibility General well-being	Unguided	Online	Individual	8.0	Post
Karimi and Aghaei (2018)	6	15	15	–	15;18	100	Waitlist	Internalizing problems	Guided	Offline	Group	18.0	Post 1 to 3 month FU
Khoramnia et al. (2020)	10	12	12	22.1 (1.08)	–;–	70.8	Waitlist	Internalizing problems Psychological flexibility Self-compassion Emotional coping	Guided	Offline	–	–	Post 1 to 3 month FU
Kocovski et al. (2019)	18	58	59	24.0 (6.72)	17;51	73.7	Waitlist	Internalizing problems Psychological flexibility Self-compassion General well-being Cognitive coping	Unguided	Offline	Individual	–	Post 1 week to 1 month FU
Krafft et al. (2019)	16	33;34	31	21.8 (–)	18;39	70.4	Waitlist	Internalizing problems Psychological flexibility General well-being	Unguided	Online	Individual	–	Post
Krafft et al. (2020)	8	55	53	20.5 (3.79)	–;–	76.5	CBT	Internalizing problems Psychological flexibility General well-being Social well-being	Unguided	Online	Individual	–	Post
Lappalainen et al. (2021)	6	83;82	84	15.3 (0.39)	15;16	51.0	Waitlist	Internalizing problems Psychological flexibility General well-being	Guided	Online & Blended	Individual	–	Post
Lappalainen et al. (2023)	4	154	80	15.0 (0.15)	–	66.7	Waitlist	Internalizing problems Psychological flexibility Self-compassion	Guided	Online	Individual	0.9	Post
Larsson et al. (2022)	10	52	61	25.1 (10.06)	–	79.0	Waitlist	Internalizing problems Other psychological problems Psychological flexibility	Unguided	Online	Individual	8.3	Post 1 week to 1 month FU
Lee et al. (2020)	4	22	17	21.0 (8.23)	12;45	87.2	Waitlist	Internalizing probles Psychological flexibility	Guided	Offline	Individual	–	Post
Levin et al. (2014)	8	37	39	18.4 (0.54)	18;20	53.9	Waitlist	Internalizing problems Psychological flexibility	Unguided	Online	Individual	–	Post
Levin et al. (2016)	33	110	118	21.6 (5.48)	18;58	76.9	Other (mental health education website)	Internalizing problems Other psychological problems Psychological flexibility	Unguided	Online	Individual	–	Post 1 week to 1 month FU 1 to 3 month FU
Levin et al. (2017)	16	40	39	20.5 (2.73)	18; –	66.0	Waitlist	Internalizing problems Externalizing problems Other psychological problems Psychological flexibility General well-being Social well-being	Unguided	Online	Individual	–	Post
Levin et al. (2019)	10	23;22	24	21.9 (5.47)	18;46	68.1	Other (daily app check-ins)	Internalizing problems Other psychological problems Psychological flexibility Social well-being	Unguided	Online	Individual	–	Post
Levin et al. (2020b)	48	46;46;45	45	22.3 (5.08)	–	72.4	Waitlist	Other psychological problems Psychological flexibility	Unguided	Online	Individual	4.5	Post 1 to 3 month FU
Levinet al. (2020a)	8	53	56	20.9 (3.76)	18;43	65.1	Other treatment (Mindfulness-based stress reduction)	Internalizing problems Other psychological problems Psychological flexibility	Unguided	Online	Individual	–	Post
Livheim et al. (2015)	10	32;15	26;17	14.6 (1.03)	12.5;17.75	100	TAU	Internalizing problems Psychological flexibility General well-being	Guided	Offline	Group	10.5	Post
Masoumian et al. (2021)	7	15	15	24.6 (3.02)	–	0	CBT	Externalizing problems Social well-being	Guided	Offline	Group	7.5	Post
Michielse et al. (2023)	1	25	28	21.5 (2.53)	16;25	75.6	Other (structured group discussion about documentaries)	Other psychological problems	Guided	Blended	Group	–	Post
Muto et al. (2011)	10	35	35	23.6 (–)	20;26	62.9	Waitlist	Internalizing problems Other psychological problems Psychological flexibility	Unguided	Offline	Individual	–	Post 1 to 3 month FU
Nissling et al. (2023)	4	27	27	16.6 (1.22)	–	82.7	Waitlist	Internalizing problems Psychological flexibility General well-being	Guided	Online	Individual	–	Post
Noormohamadi et al. (2022)	4	10	10	25.0 (2.77)	18;28	–	–	Other psychological problems Cognitive coping	Guided	Offline	Individual	8.3	Post 1 week to 1 month FU
Ong et al. (2019)	16	26	23	25.4 (12.30)	–	74.0	Waitlist	Other psychological problems Psychological flexibility Self-compassion General well-being Cognitive coping	Guided	Offline	Individual	8.7	Post 1 week to 1 month FU
Othman et al. (2023)	8	34	34;34	23.8 (–)	19;29	84.0	CBT Waitlist	Internalizing problems	Guided	Offline	Group	4.0	Post 1 to 3 month FU
Pahnke et al. (2014)	10	15	13	16.5 (2.00)	13;21	25.0	Waitlist	Internalizing problems Externalizing problems Social well-being	Guided	Offline	Group	9.0	Post 1 to 3 month FU
Petersen et al. (2023)	10	13	13	15.7 (1.60)	–	73.1	Waitlist	Internalizing problems Psychological flexibility General well-being	Guided	Offline	Group	8.0	Post 1 week to 1 month FU
Pitil and Ghazali (2023)	6	52	50	21.2 (1.63)	–	57.8	Waitlist	Cognitive coping	Guided	Online	Group	–	Post 1 to 3 month FU
Puolakanaho et al. (2019)	2	165	84	15.27 (0.39)	–	51	Waitlist	Internalizing problems	Guided	Online	Individual	–	Post
Räsänen et al. (2016)	11	33	35	24.3 (3.28)	–	85.3	Waitlist	Internalizing problems Other psychological problems Psychological flexibility General well-being	Guided	Blended	Individual	10.0	Post
Shabani et al. (2019)	20	22	22;25	15.0 (1.47)	–	44.9	CBT Waitlist	Internalizing problems Psychological flexibility	Guided	Offline	Group	–	Post 1 to 3 month FU
Talaeizadeh (2020)	4	15	15;15	14.9 (–)	–	100	CBT Waitlist	Internalizing problems General well-being	Guided	Offline		12.0	Post
Uğur and Koç (2021)	2	13	13	20.0 (–)	–	50	Waitlist	Internalizing problems	Guided	Offline	Group	5.0	Post 1 to 3 month FU
Uysal et al. (2024)	3	75	75	18.8 (0.72)	17;20	82	CBT	Internalizing problems Cognitive coping	Guided	Online	Group	12.0	Post
Vakilian et al. (2019)	4	22	22	22.3 (1.45)	–	100	TAU	Internalizing problems General well-being	Guided	Offline	Group	7.0	Post 1 week to 1 month FU
Vakilian et al. (2023)	2	33	33	25.3 (5.1)	–	100	TAU	Internalizing problems	Guided	Offline	Group	8.0	Post 1 week to 1 month FU
Wahyun et al. (2019)	1	29	29	18.5 (–)	18;19	96.6	Waitlist	General well-being	Guided	Offline	Group	12.5	Post
Wang et al. (2017)	6	26	26	21.1 (2.42)	18;30	43.6	CBT	Internalizing problems General well-being	Guided	Offline	Group	24.0	Post 1 to 3 month FU
Watson et al. (2023)	12	41	40	24.3 (6.77)	18;44	100	Waitlist	Internalizing problems Externalizing problems General well-being Cognitive coping	Unguided	Online	Individual	–	Post
Yadavaia et al. (2014)	12	34	44	20.5 (4.33)	18; –	74.0	Waitlist	Internalizing problems Psychological flexibility Self-compassion	Guided	Offline	Group	6.0	Post 1 to 3 month FU
Zemestani and Mozaffari (2020)	8	30	30	24.5 (4.21)	–	73.3	Other treatment (psychoeduction about depression)	Internalizing problems Psychological flexibility General well-being Emotional coping	Guided	Offline	Group	12.0	Post 1 to 3 month FU
Zemestani et al. (2022)	10	24	47	15.2 (0.4)	15;18	100	Waitlist	Internalizing problems Emotional coping Cognitive coping	Guided	Online	Group	7.0	Post 1 week to 1 month FU
Zhao et al. (2022)	4	95	87	21.8 (1.33)	–	52.7	Waitlist	Internalizing problems Other psychological problems	Unguided	Online	Individual	3.0	Post 1 to 3 month FU

FU = Follow-up

## Risk of Bias

Table 2 presents the risk of bias scores for all studies across the six domains, as well as the percentage of studies with low, unclear, and high risk in each domain. The most common source of bias was the lack of blinding of participants, personnel, and outcome assessors, which is expected in studies evaluating the efficacy of psychological interventions. Notably, 16.7% of the studies had a high risk of bias in the selective reporting domain and the percentages of studies with unclear risk in the remaining domains ranged from 31.8 to 74.2%. Moreover, 95.5% of the studies had a high or unclear risk of bias on at least one other domain than a lack of blinding. This indicates the presence of other potential sources of bias beyond blinding.

**Table 2:** Risk of Bias in the Included Randomized Controlled Trials

Authors (year)	Random sequence generation	Allocation concealment	Blinding of participants and personnel	Blinding of outcome assessment	Incomplete outcome data (post-measurement)	Incomplete outcome data (follow-up 1)	Incomplete outcome data (follow-up 2)	Selective outcome reporting
Asale et al. (2021)	–	?	–	–	?			?
Van Aubel et al. (2020)	+	+	–	–	+	?	?	?
Azadeh et al. (2016)	?	?	–	–	?			?
Barreto and Gaynor (2023)	+	?	–	–	+	+		?
Bernal-Manrique et al. (2020)	+	+	–	–	+			?
Butryn et al. (2011)	?	?	–	–	?	?		?
Değerli and Odacı (2024)	?	?	–	–	?	?	?	?
Dereix-Calonge et al. (2019)	+	+	–	–	+			?
Ditton et al. (2023)	+	+	–	–	+			+
Ekeanya et al. (2023)	?	?	–	–	?			?
El-Ashry et al. (2023)	+	+	–	–	?	?		+
Enayati et al. (2018)	?	?	–	–	?	?		?
Engström et al. (2022)	+	?	–	–	+	+		–
Fabricant et al. (2013)	?	?	–	–	+			?
Fang et al. (2023)	?	?	–	–	+			?
Frögéli et al. (2016)	?	?	–	–	+	+		?
Ghasemi et al. (2023)	+	?	–	–	?	?		?
Gloster et al. (2017)	?	?	–	–	–	–		?
Van der Gucht et al. (2017)	+	+	–	–	?	?		?
Hashemipoor et al. (2021)	?	?	–	–	?	?		?
Hayes et al. (2011)	+	+	–	–	+	?		?
Ito and Muto (2020)	+	+	–	–	+	+		+
Karaaziz et al. (2023)	?	?	–	–	?	?		?
Karekla et al. (2022)	?	?	–	–	+			–
Karimi and Aghaei (2018)	?	?	–	–	+	+		?
Khoramnia et al. (2020)	+	?	–	–	–	–		–
Kocovski et al. (2019)	+	?	–	–	+	+		–
Krafft et al. (2019)	+	?	–	–	+			?
Krafft et al. (2020)	+	+	–	–	+			–
Lappalainen et al. (2021)	+	+	–	–	+			–
Lappalainen et al. (2023)	+	+	–	–	?			–
Larsson et al. (2022)	+	+	–	–	+	+		+
Lee et al. (2020)	+	?	–	–	+			?
Levin et al. (2014)	?	?	–	–	+			?
Levin et al. (2016)	+	+	–	–	+	+	+	?
Levin et al. (2017)	+	+	–	–	+			?
Levin et al. (2019)	+	+	–	–	+			?
Levin et al. (2020b)	+	+	–	–	?	?		?
Levin et al. (2020a)	+	+	–	–	+			?
Livheim et al. (2015)—Australia	+	+	–	–	+			?
Livheim et al. (2015)—Sweden	+	?	–	–	+			?
Masoumian et al. (2021)	?	?	–	–	?			–
Michielse et al. (2023)	+	+	–	–	?			?
Muto et al. (2011)	+	+	–	–	+	+		?
Nissling et al. (2023)	+	?	–	–	+			?
Noormohamadi et al. (2022)	?	?	–	–	?	?		?
Ong et al. (2019)	+	+	–	–	+	+		?
Othman et al. (2023)	?	?	–	–	?	?		?
Pahnke et al. (2014)	+	+	–	–	+			?
Petersen et al. (2023)	?	?	–	–	+	+		?
Pitil and Ghazali (2023)	?	?	–	–	+	+		?
Puolakanaho et al. (2019)	?	+	–	–	+			?
Räsänen et al. (2016)	?	+	–	–	+	+		?
Shabani et al. (2019)	+	?	–	–	+	+		–
Talaeizadeh (2020)	?	?	–	–	+			?
Uğur and Koç (2021)	+	+	–	–	+	+		?
Uysal et al. (2024)	?	?	–	–	?			?
Vakilian et al. (2019)	?	?	–	–	+	+		+
Vakilian et al. (2023)	+	+	–	–	+	+		–
Wahyun et al. (2019)	?	?	–	–	?			?
Wang et al. (2017)	?	?	–	–	?	?		?
Watson et al. (2023)	?	–	–	–	+			+
Yadavaia et al. (2014)	+	?	–	–	+	+	+	?
Zemestani and Mozaffari (2020)	+	+	–	–	?	?		–
Zemestani et al. (2022)	+	?	–	–	+	+		?
Zhao et al. (2022)	–	+	–	–	+	+		?
% studies with low risk	56.1	42.4	0	0	65.2	54.1	50.0	9.1
% studies with unclear risk	40.9	56.1	0	0	31.8	40.5	50.0	74.2
% studies with high risk	3.0	1.5	100	100	3.0	5.4	0	16.7

+  = low risk; ? = unclear; −  = high risk

## Overall Effect

The overall effect size on psychopathology, ACT related processes, well-being and coping (expressed in Hedges’ g) was 0.72 with a standard error of 0.10. The overall effect was significant (*t*(583) = 7.55, *p* < .001, CI = 0.53—0.91) and can be regarded as a moderate effect. We found significant variance at both the within study level (level 2) as the between study level (level 3). Particularly, the fit of the full model was significantly better than the fit of the models where the within study variance or the between study variances were fixed to zero (LRT_within_ = 452.64, *p* < .0001; LRT_between_ = 267.51, *p* < .0001). This implies that there is more variability in effect sizes (within and between studies) than can be expected based on sampling variance alone. Therefore, meta-regression analyses can be performed in order to examine variables that may explain within- and/or between-study variance. Using the formula of Cheung (2014) we found that 6.25 percent of the total variance can be attributed to variance at level 1 (i.e., the typical within-study sampling variance); 26.58 percent of the total variance can be attributed to differences between effect sizes within studies at level 2 (i.e., within-study variance); and 67.17 percent of the total variance can be attributed to differences between studies at level 3 (i.e., between-study variance; *I*^*2*^ statistic).

The quality of evidence of the overall effect was graded as very low. We downgraded for risk of bias (95.5% of the studies had a high or unclear risk of bias on at least one other domains than a lack of blinding), inconsistency in findings (i.e., significant heterogeneity between effect sizes) and publication bias (Egger’s regression test was significant and trim and fill analyses showed that effect sizes were missing on the right side of the distribution; see last paragraph of the results section). We did not downgrade for indirectness and imprecision (as the confidence interval of the effect size was not too wide).

## Meta-Regression Analyses

The results of the separate meta-regression analyses, including the effect sizes for each (sub) outcome, timing of assessment, category of our intervention characteristics, control group and category of our sample characteristics, are presented in Table 3. We were unable to conduct meta-regression analyses for "treatment integrity" and "comorbidity", since treatment integrity was reported in only 6 studies (9.2%) and comorbidity was explicitly mentioned in only 9 studies (13.8%).

**Table 3:** Meta-Regression Results: Separate Analyses for Each Predictor

Predictors	*N* studies	*N* ES	Mean (Min–Max)	*B*_*0*_ (95% CI)	*t*_*0*_	*B*_*1*_ (95% CI)	*t*_*1*_	*F*(df_1,_ df_2_)	*p*	Within study variance Between study variance
*Type of (sub) outcome*										
Type of outcome								*F*(3, 580) = 0.05	.983	0.212*** 0.537***
Psychopathology^1^	56	260	–	**0.72** **(0.53;0,92)**	7.25***					
ACT related processes	42	199	–	**0.73** **(0.53;0.94)**	7.06***	0.01 (–0.10;0.12)	0.17			
Well-being	31	81	–	**0.71** **(0.48;0.94)**	6.13***	–0.01 (–0.17;0.15)	–0.17			
Coping	15	44	–	**0.69** **(0.42;0.97)**	4.95***	–0.03 (–0.26;0.19)	–0.27			
Type of psychopathology								*F*(2, 257) = 2.21	.111	0.234*** 0.393***
Internalizing^1^	49	193	–	**0.59** **(0.40;0.78)**	6.05***					
Externalizing	5	13	–	0.41 (–0.00;0.83)	1.96	–0.18 (–0.57;0.22)	–0.88			
Other	19	54	–	**0.78** **(0.53;1.04)**	6.00***	0.19 (–0.03;0.41)	1.72			
Type of ACT related process								*F*(1, 197) = 2.03	.156	0.041*** 0.249***
Psychological flexibility^1^	42	187	–	**0.57** **(0.40;0.73)**	6.73***					
Self-compassion	6	12	–	**0.75** **(0.46;1.04)**	5.10***	0.18 (–0.07;0.44)	1.43			
Type of well-being								*F*(1,79) = 1.60	.210	0.127* 2.936***
General well-being^1^	25	54	–	**0.92** **(0.28;1.55)**	2.87**					
Social well-being	12	27	–	**1.16** **(0.47;1.85)**	3.36***	0.25 (–0.14;0.64)	1.26			
Type of coping								*F*(1,42) = 2.25	.141	0.558*** 0.000
Emotional coping^1^	6	10	–	**1.25** **(0.72;1.77)**	4.82***					
Cognitive coping	11	34	–	**0.81** **(0.53;1.08)**	5.85***	–0.44 (–1.03;0.152)	–1.50			
*Timing of assessment*								*F*(3,574) = 1.12	.340	0.200*** 0.505***
Post intervention^1^	65	382	–	**0.70** **(0.52;0.89)**	7.43***					
1 week to 1 month follow-up	14	63	–	**0.73** **(0.49;0.97)**	5.97***	0.03 (–0.15;0.21)	0.33			
1 to 3 months follow-up	19	107	–	**0.80** **(0.58;1.01)**	7.17***	0.09 (–0.05;0.23)	1.31			
3 to 12 months follow-up	4	26	–	**0.54** **(0.22;0.85)**	3.33***	–0.17 (–0.43;0.10)	–1.23			
*Intervention characteristics*										
Guidance			–					***F*(1,580) = 5.20**	**.023**	0.209*** 0.495***
Guided^1^	49	357	–	**0.84** **(0.63;1.05)**	7.83***					
Unguided	15	225	–	0.35 (–0.02;0.72)	1.89	–**0.49** **(**–**0.91;** –**0.07)**	–2.28*			
Delivery format								***F*(2,579) = 4.61**	**.010**	0.210*** 0.457***
Offline^1^	38	302	–	**0.92** **(0.69;1.15)**	7.85***					
Online	23	249	–	**0.48** **(0.19;0.76)**	3.31***	–**0.45** **(**–**0.81;** –**0.08)**	–2.40*			
Blended	4	31	–	0.08 (–0.49;0.66)	0.28	–**0.84** **(**–**1.46;** –**0.22)**	–2.67**			
Therapy format								***F*(1,562) = 7.11**	**.008**	0.206*** 0.480***
Group^1^	33	253	–	**0.92** **(0.67;1.18)**	7.15***					
Individual	28	311	–	**0.42** **(0.15;0.69)**	3.09**	–**0.50** **(**–**0.87;** –**0.13)**	–2.67**			
Duration (hours)	42	352	9.37 (0.8–24.0)	**0.86** **(0.55;1.17)**	5.49***	0.04 (–0.02;0.09)	1.23	*F*(1,350) = 1.52	.218	0.364*** 0.924***
N of PF processes	65	584	4.67 (0–6)	**0.72** **(0.53;0.91)**	7.50***	0.05 (–0.04;0.14)	1.10	*F*(1,582) = 1.21	.273	0.210*** 0.537***
*Type of control group*								*F*(4,567) = 18.12	< .001	0.174*** 0.671***
Waitlist^1^	43	394	–	**0.93** **(0.69;1.17)**	7.64***					
TAU	5	28	–	**0.81** **(0.05;1.56)**	2.10*	–0.13 (–0.92;0.67)	–0.31			
CBT	7	40	–	–0.24 (–0.63;0.14)	–1.25	–**1.18** **(**–**1.52;** –**0.84)**	–6.79***			
Other treatment	6	30	–	–0.09 (–0.50;0.32)	–0.43	–**1.02** **(**–**1.40;** –**0.64)**	–5.31***			
Other	8	80	–	0.57 (–0.01;1.14)	1.94	–0.36 (–0.98;0.25)	–1.17			
*Sample characteristics*										
Mean age	63	568	20.4 (14.5–25.4)	**0.72** **(0.52;0,92)**	7.15***	–0.00 (–0.05;0.05)	–0.10	*F*(1,566) = 0.01	.922	0.213*** 0.567***
Gender (% female)	63	572	72.7 (0–100)	**0.66** **(0.49;0.83)**	7.64***	**0.01** **(0.00;0.02)**	2.27*	***F*(1,570) = 5.16**	**.024**	0.170*** 0.416***
Target group								*F*(3,580) = 1.11	.344	0.210*** 0.529***
Non-clinical^1^	26	256	–	**0.58** **(0.29;0.87)**	3.93***					
Subclinical	26	240	–	**0.75** **(0.45;1.05)**	4.93***	0.17 (–0.25;0.59)	0.79			
Clinical	11	82	–	**1.05** **(0.59;1.51)**	4.52***	0.47 (–0.07;1.01)	1.70			
Mixed	2	6	–	0.36 (–0.74;1.46)	0.65	–0.22 (–1.36;0.91)	–0.39			

Significant results are highlighted in bold

B_0_, mean effect size Hedges’ g; CBT, cognitive behavioural therapy; CI, confidence interval; B_1_, estimated regression coefficient; F-value, omnibus test of regression coefficients; N of PF processes; number of psychological flexibility processes that are described in the study; N ES, number of effect sizes; N samples, number of independent samples; p, *p*-value of omnibus test; TAU, treatment as usual; *t*-values, difference in mean g with zero

^1^Is reference category

^*^p < .05; **p < .01; ***p < .001

First, concerning the type of (sub) outcomes, significant effects were found for all main (Hedges’ g ranged from 0.69 to 0.73) and most sub outcomes (Hedges’ g ranged from 0.57 to 1.25). ACT did only not outperform control conditions with regard to externalizing symptoms (Hedges’ g was 0.41). No differences in effect sizes were found based on the type of main outcome, type of psychopathology, type of ACT-related process, type of well-being, and type of coping. Second, the meta-regression analysis for timing of assessment showed no significant differences between assessment points and indicated significant effects at all assessment points. Third, with regard to the intervention characteristics we found significant meta-regression analyses for guidance, type of delivery format and type of therapy format. Particularly, significant effects were found for studies investigating guided, offline, online, group and individual ACT, but not for studies examining blended and unguided ACT. Studies investigating guided ACT interventions yielded larger effects compared to studies examining unguided ACT interventions. Compared to studies investigating offline ACT, studies examining online or blended ACT yielded smaller effects. Studies investigating ACT as a group intervention demonstrated larger effects compared to studies investigating ACT as an individual intervention. No differences in effect sizes were found regarding duration of the intervention and number of psychological flexibility processes. Fourth, regarding the type of control group, we found significant effects in studies comparing ACT with a waitlist and TAU condition and no significant effects in studies comparing ACT with CBT, another treatment (e.g., REBT) or other control group. Studies comparing ACT with CBT or another treatment yielded smaller effects than studies that compared ACT with a waitlist. Last, concerning the sample characteristics we found a significant meta-regression analysis for gender. Specifically, a higher proportion of females in studies was associated with larger effects. The meta-regression analyses of mean age and target group were nonsignificant. Notably, we did find significant effects for studies conducted in non-clinical, subclinical and clinical samples but not for studies conducted in mixed samples.

We inspected if results remained significant when testing all significant predictors in a single model. The VIF values for all predictors ranged from 1.04 (for other treatment) to 3.29 (for unguided ACT), indicating no multicollinearity issues. Results are presented in Table 4. The omnibus test showed a significant result (*F*(9, 534) = 8.99, *p* < .001), indicating that at least one of the predictors yielded a significant result. Based on the results, we can conclude that CBT (compared to waitlist), other treatments (compared to waitlist), and other control groups (compared to waitlist) remained significant predictors in the model. Guidance, delivery format, therapy format, and gender were no longer significant predictors. These results indicate that, when controlling for other significant predictors, the effect size for comparing ACT to waitlist conditions was not significantly different from the effect size for comparing ACT to TAU, but it was larger than the effect sizes for comparisons between ACT and CBT, ACT and other treatments, and ACT and other control conditions. After conducting the meta-regression including all significant predictors, we still found significant variance at both the within study level as the between study level. Particularly, the fit of the full model was significantly better than the fit of the models where the within study variance or the between study variances were fixed to zero (LRT_within_ = 245.09, *p* < .0001; LRT_between_ = 165.58, *p* < .0001).

**Table 4:** Meta-Regression Results: All Significant Predictors in One Model

Predictors	*B*_*1*_ (95% CI)	*t*_*1*_	*p*
*Guidance (guided*^*1*^*)*			
Unguided	–0.08 (–0.61;0.46)	–0.28	.779
*Delivery format (offline*^*1*^*)*			
Online	–0.11 (–0.53;0.32)	–0.48	.632
Blended	–0.48 (–1.08;0.12)	–1.58	.115
*Therapy format (group*^*1*^*)*			
Individual	–0.41 (–0.88:0.05)	–1.74	.082
*Control group (waitlist*^*1*^*)*			
TAU	–0.26 (–0.91;0.39)	–0.79	0.432
CBT	–1.03 (–1.35; –0.71)	–6.30	< .001
Other treatment	–0.86 (–1.21; –0.52)	–4.90	< .001
Other	–0.73 (–1.31; –0.14)	–2.43	.015
*Gender*	0.01 (–0.00;0.02)	1.66	.098

CBT, cognitive behavioural therapy; CI, confidence interval; B_1_, estimated regression coefficient; p, *p*-value of t-test; TAU, treatment as usual; *t*-values, difference in mean g with zero

^1^reference category

## Sensitivity Analyses

We conducted two sensitivity analyses to check the robustness of the overall effect and meta-regression analyses. First, we conducted a sensitivity analysis removing seven outliers (i.e., effect sizes with z values larger than 3.29 or smaller then -.3.29; Tabachnik & Fidell, 2013). This analysis produced an overall effect of 0.64 (Hedges’ g) with a standard error of 0.08 (*t*(576) = , 8.50, *p* < .001, CI = 0.49—0.79). This overall effect falls within the confidence interval of the original effect size, suggesting that the outliers did not have a significant impact on our overall results. While our overall conclusions remained consistent, there were some changes in the outcomes of the meta-regression analyses. Specifically, the effect sizes for studies investigating the effects of ACT on externalizing symptoms (B_0_ = 0.43, 95% CI = 0.09–0.78, T_0_ = 2.48, *p* = .014), examining unguided ACT interventions (B_0_ = 0.35, 95% CI = 0.06–0.64, T_0_ = 2.39, *p* = .017) and those comparing ACT to other control conditions (e.g., educational interventions) (B_0_ = 0.49, 95% CI = 0.05–0.93, T_0_ = 2.18, *p* = .030) were now significantly different from zero. Moreover, meta-regression analyses now also showed that type of psychopathology was a significant predictor (F(2, 254) = 3.92, *p* = .021). Specifically, studies investigating ACT’s effect on other outcomes (B_0_ = 0.79) showed higher effect sizes compared to studies focusing on internalizing (B_0_ = 0.56, B_1_ = 0.23, T_1_ = 2.46, *p* = .015) and externalizing symptoms (B_0_ = 0.43, B_1_ = 0.36, T_1_ = 2.13, *p* = .034). Furthermore, while the type of target group was not a significant predictor overall (F(3,573) = 2.03, *p* = .109), studies in clinical samples (B_0_ = 1.03) exhibited larger effects compared to studies in non-clinical samples (B_0_ = 0.53, B_1_ = 0.50, T_1_ = 2.35, *p* = .019). Results regarding the other meta-regression analyses remained similar. Since the sensitivity analysis identified two additional predictors (type of psychopathology and target group) for effect size heterogeneity, these predictors were added to the meta-regression analysis with all significant predictors. Results are presented in Appendix C. The analysis without outliers suggests that, in addition to the type of control group, factors as type of psychopathology, delivery format, and gender may also uniquely contribute to heterogeneity in effect sizes. Specifically, studies examining ACT’s impact on internalizing and externalizing symptoms, studying blended ACT (only four studies) and including a higher proportion of males showed smaller effects compared to studies focusing on other outcomes, examining offline ACT and including a higher proportion of females.

Second, we conducted a sensitivity analyses to explore whether the results would vary if the third level represented the variance in effect sizes between samples instead of between studies, as was the case in the main analyses. There were 67 independent samples and 65 studies. This analysis produced an overall effect of 0.71 (Hedges’ g) with a standard error of 0.09 (*t*(583) = ,7.66 *p* < .001, CI = 0.53—0.89) which also lies within the confident interval of the original effect size. Moreover, conclusions with regard to the meta-regression analyses remained unchanged.

## Publication Bias

**Fig. 2:**
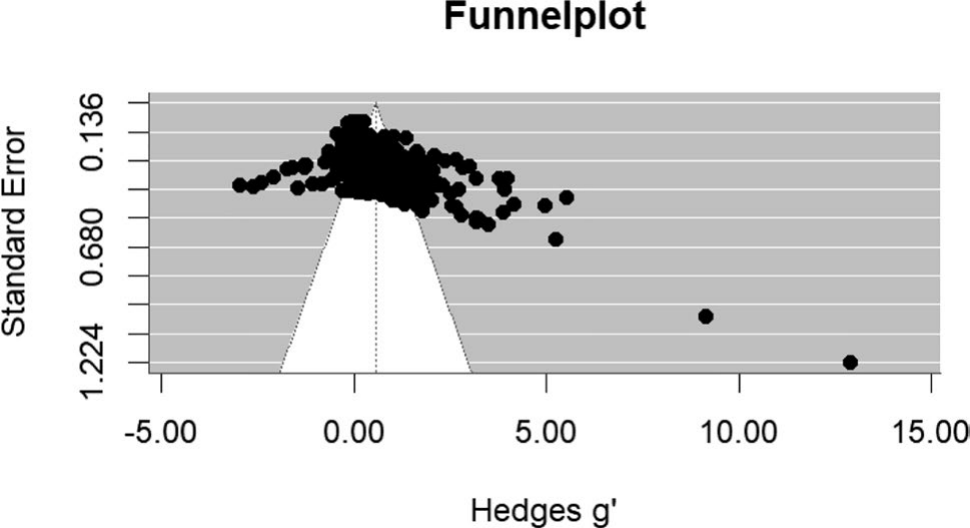
Funnel Plot

**Fig. 3:**
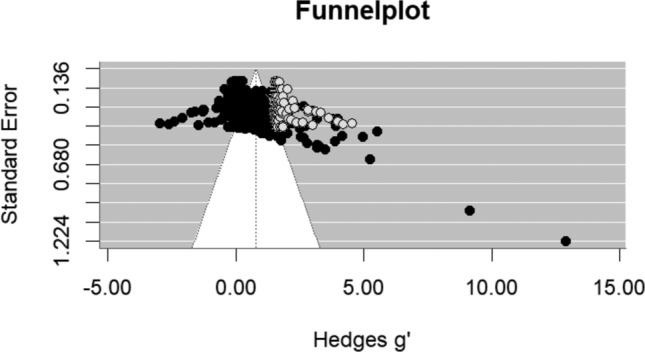
Trim and Fill analyses on the Right Side of the Distribution

The funnel plot is presented in Fig. 2. According to Egger’s regression test, the distribution of effect sizes appeared to be asymmetrical (*B* = -0.63, z = -6.04, *p* < .001) indicating there was risk of publication bias. Trim and fill analyses estimated that zero effect sizes were missing on the left side of the distribution and 120 effect sizes were missing at the right side of the distribution (see Fig. 3). Imputing these effect sizes on the right side resulted in an adjusted Hedges g’ of 0.78 with a standard error of 0.04 and a 95% confidence interval between 0.71 and 0.85.

## Discussion

The current meta-analysis integrated the findings of 65 RCTs (*n* = 5283) investigating the efficacy of ACT for TAY on psychopathology (i.e., internalizing, externalizing and other psychological problems), ACT related processes (i.e., psychological flexibility and self-compassion), well-being (i.e., general and social well-being) and coping (i.e., emotional and cognitive coping). Overall, we found a moderate effect (Hedges’s g = 0.72) of ACT compared to the control conditions on psychopathology, ACT related processes, well-being and coping. The quality of the evidence was very low due to a relatively high risk of bias in the selected studies, considerable heterogeneity in effect sizes and risk of publication bias.

To explain the heterogeneity in effect sizes, we conducted several meta-regression analyses, including type of (sub) outcome, timing of assessment, various intervention characteristics, type of control group and several sample characteristics. First, due to mixed or insufficient findings in earlier research, the meta-regression analyses regarding the type of (sub) outcome(s) were conducted in an exploratory manner. In our meta-analysis, ACT appeared effective across all types of main outcome (i.e., psychopathology, ACT related processes, well-being and coping) and most types of sub outcomes (i.e., internalizing and other psychological problems, psychological flexibility and self-compassion, general and social well-being and emotional and cognitive coping). ACT did solely not outperform control conditions with regard to externalizing symptoms, but this was likely due to limited statistical power (i.e., externalizing symptoms was only included as an outcome in 5 studies). Moreover, after removing outliers, we did find a significant effect of ACT on externalizing symptoms. Overall, no differences in effect sizes were found across studies examining different types of main and sub-outcomes. These results indicate that, aside from increasing TAY’s psychological flexibility (i.e., the primary goal of ACT), ACT also has an (indirect) effect on their psychological problems, well-being and coping. This finding is in line with earlier meta-analyses in younger populations, which have demonstrated the efficacy of ACT in reducing psychopathology (Fang & Ding, 2020; Perkins et al., 2022; Wang & Fang, 2023) and enhancing psychological flexibility processes (Parmar et al., 2021; Wang & Fang, 2023) and well-being (Fang & Ding, 2020; Howell & Passmore, 2019). Moreover, it aligns with the transdiagnostic nature of ACT and its theoretical framework, which proposes that improving clients’ psychological flexibility can lead to a reduction in their psychological problems and an improvement in their quality of life (Hayes et al., 2006).

Second, ACT was found to be effective at both post-intervention and various follow-up assessments. In line with A-Tjak et al. (2015) and Powers et al. (2009), there were no differences between effect sizes at the different assessment points. This suggests that ACT is both effective at the short and long term. However, results should be interpreted cautiously because the majority of studies had short follow-up periods (i.e., less than three months).

Third, meta-regression analyses for several intervention characteristics were conducted. We found a significant effect for guided ACT interventions but not for unguided ACT interventions. In line with previous meta-analyses (French et al., 2017; Spijkerman et al., 2016; Thompson et al., 2021), studies investigating guided ACT (guided therapy and self-help interventions with additional guidance) yielded larger effects than studies examining unguided ACT (self-help books, apps or websites). Although this might suggest that unguided ACT interventions are not effective, the results should be interpreted with caution. Predominantly, as the effect of unguided ACT was not robust (i.e., with outliers, unguided ACT did not outperform controls but without outliers it did). Moreover, when controlling for other significant predictors, the difference in effect sizes between guided and unguided ACT became insignificant. In addition, in most of the included studies, unguided ACT was compared with waitlist conditions and solely examined in subclinical and non-clinical samples. This could have potentially lead to an overestimation (e.g., larger effect sizes when compared to waitlist than active control conditions) or underestimation (e.g., smaller effect sizes in nonclinical populations as there is less room for improvement than in clinical samples) of their effects.

Based on meta-analyses in adult samples, we did not expect to find differences in effect sizes between studies investigating various delivery formats (Di Sante et al., 2022; French et al., 2017; Han & Kim, 2022). However, in our meta-analysis, we found significant effects for both offline and online ACT interventions but not for blended ACT interventions. Moreover, studies investigating offline ACT yielded larger effect sizes compared to studies examining online and blended ACT. Nonetheless, these findings should also be interpreted cautiously. Specifically, when all significant predictors were analyzed together, the differences between offline, online and blended ACT were no longer significant. Additionally, the effect of blended ACT may have been underpowered, as it was examined in only four studies. Furthermore, also here, in the included studies, the online and blended interventions were predominantly investigated in subclinical and non-clinical samples, limiting generalizability to clinically referred TAY.

Regarding therapy format, we found both group and individual ACT to be more effective compared to control conditions. In contrast with earlier studies (Öst, 2014; Perkins et al., 2022; Ruiz, 2012) studies investigating group ACT showed higher effect sizes than studies investigating individual ACT. Notwithstanding, the difference was no longer significant when controlling for other significant predictors, suggesting that this result should be interpreted with more caution. The meta-regression analyses of duration of the intervention and the number of psychological flexibility processes were not significant. Although results from earlier meta-analyses were mixed, our results suggest that also shorter ACT interventions might have beneficial effects. The results concerning the number of psychological flexibility processes addressed in the intervention should be interpreted with caution. We relied on intervention descriptions in the article (instead of the actual intervention protocols) to code the number of psychological flexibility processes. Hence, some psychological flexibility processes may be coded differently than the actual intervention. Due to insufficient reporting, the meta-regression regarding treatment integrity could not be conducted.

Fourth, concerning the type of control group, we found that ACT was more effective than waitlist and TAU, but equally effective compared to CBT, other treatments (e.g., REBT) and other control conditions (e.g., educational intervention). This result was consistent with earlier meta-analyses in both adult (Gloster et al., 2020) and youth (Fang & Ding, 2020) populations and suggests that ACT can be regarded as an effective treatment for various problems in TAY. The finding that ACT was equally effective compared to other control conditions (e.g., educational interventions) was somewhat unexpected. This may be due to the fact that this result was based on only eight studies, all examining brief ACT interventions (ranging from one to six sessions) in primarily non-clinical samples. Moreover, after removing outliers we did find a significant effect size for studies comparing ACT to other control conditions.

Fifth, concerning the sample characteristics, the meta-regression of the studies’ mean age was not significant. This is consistent with some earlier meta-analyses (Öst, 2014; Perkins et al., 2022) and may suggest that ACT works equally well across different ages in this developmental period. Consistent with the meta-analysis of Öst (2014), studies with more females showed larger effects than studies with fewer females. However, when all significant predictors were tested simultaneously, this gender effect became insignificant. Hence, more research into gender effects are needed. Finally, ACT appeared effective across non-clinical, subclinical and clinical samples. Also, the type of target group was not a significant predictor. Hence, in line with other meta-analyses (Bai et al., 2020; Dawson et al., 2022; Öst, 2014; Sun et al., 2022), the effects of ACT might be similar across various target groups. Due to insufficient available information, we could not conduct the meta-regression analysis of comorbidity.

There are some limitations of the current meta-analysis that should be discussed. Some study limitations are driven by limitations of the included studies. First, there was a considerable risk of bias in domains other than the lack of blinding (which is unavoidable in studies evaluating the efficacy of psychological interventions). We recommend future efficacy researchers on ACT to enhance research quality by performing intent-to-treat analyses (in 37.9% of the studies the type of analysis was unclear or completer only analyses were performed), pre-registering their studies (74.2% of the studies were not pre-registered) and more elaborate reporting on the randomization procedures and reasons for drop-out. Second, 66.2% of the studies compared ACT with a waitlist condition, 55.4% included 30 or fewer participants per condition and only 6.2% conducted follow-up assessments after three months. Due to the use of waitlist controls and small sample sizes, the overall effect of ACT may be somewhat overestimated. These factors also likely contributed to the presence of extremely high (i.e., Hedges’ g > 2.0) effect sizes in our meta-analysis. Additionally, due to the limited follow-up data, conclusions about long-term effects should be interpreted with caution. To draw more reliable conclusions, future studies should compare ACT with active control conditions, include larger sample sizes and incorporate longer follow-up periods. Third, not all relevant study aspects were reported in detail. Hence, it was not possible to conduct some of the relevant meta-regressions. We therefore encourage more detailed reporting of the content of the intervention (e.g., which psychological flexibility processes were targeted) and the level of comorbidity of participants (i.e., do participants suffer from one or more disorders). Furthermore, we would like to stimulate intervention researchers to assess treatment integrity (assessed in only 9.2% of the studies) as measuring this is essential for validating findings, addressing implementation challenges and improving transparency in research and clinical practice. Last, the studies included in this meta-analysis were not always generalizable to a broader population of TAY. Specifically, no studies explicitly included a sample of TAY aged 15 to 25 (samples either comprised adolescents below the age 18 or adults above the age of 18), only 6.2% of the studies investigated the effects of blended ACT and solely 7.7% of the studies examined the effects of ACT on externalizing symptoms. In addition, 82.1% of studies were performed in university, college of high school samples and 81.5% of the studies were performed in Anglosphere, Western or Southern Asian countries. Moreover, the effects of alternative forms of ACT (i.e., self-help, online and brief ACT interventions) were predominantly studied in non-clinical or subclinical samples. Therefore, to improve generalizability, more research is needed in TAY specifically, on blended forms of ACT, on externalizing symptoms, in lower-educated samples, and in countries beyond the Anglosphere, Western, and Southern Asian countries. Furthermore, future studies should investigate the effects of alternative forms of ACT in clinically referred TAY.

The meta-analysis itself also had some limitations. First, there was significant heterogeneity in effect sizes, which was only partly explained by the meta-regression analyses we conducted. It might be that other predictors, such as therapeutic alliance or therapist competence, could better explain the heterogeneity in effect sizes than the predictors we included in the study. Since most studies do not yet measure or report these concepts, we encourage researchers to incorporate and report these metrics more frequently in their efficacy studies. Second, we followed the procedure of Assink and Wibbelink (2016) and first conducted separate meta-regression analyses for each predictor and then combined all significant predictors together in a single model. By doing so we could examine if significant predictors were potentially confounded by other significant predictors. However, to prevent overfitting of the model, non-significant predictors were not tested together with other predictors. Hence, we are not sure if these effects were confounded by other variables. Third, risk of bias was assessed with version one of the Cochrane risk-of-bias tool. Unlike the second version, this first version may have unfairly categorized therapy trials as high risk due to the lack of blinding. For example, a review on pediatric OCD by Cevin et al. (2024) using the second version of the tool rated the RCT of March (2004) as low risk, whereas an earlier review by Uhre et al. (2020) using the first version of the tool rated the same RCT as high risk. Hence, it is important to consider that using this first version of the tool could have led to an overestimation of risk of bias. Finally, we only included English studies and did not search for unpublished data and grey literature. This might have limited the generalizability of our findings and increased the risk of publication bias.

Notwithstanding the limitations, the study also had some important strengths. To our knowledge the current meta-analysis was the first to investigate the efficacy of ACT for TAY. Investigating the effects of ACT for this specific age group is relevant as TAY are more likely to experience psychological problems compared to other age groups (Whiteford et al., 2013). Moreover, the transdiagnostic nature of ACT along with its focus on autonomy and identity development makes ACT a potentially suitable intervention for this age group. Additionally, in our meta-analysis we included a broad range of studies that were conducted in diverse populations of TAY (e.g., various countries, target groups and types of problems), studied different forms of ACT (e.g., guided/unguided, online/blended/offline, group/individual) and measured various outcomes (i.e., psychopathology, ACT related processes, well-being and coping). This diversity improves the generalizability of the findings and allowed us to include 65 RCTs, which was significantly more than previous meta-analyses on ACT in adolescents and/or young adults (i.e., including fewer than 15 RCTs). This large and diverse sample of studies not only increased statistical power but also enabled us to numerous relevant meta-regression analyses. Finally, we performed a multilevel meta-analysis, which has advantages over more traditional meta-analyses. Specifically, it considers the dependency of multiple effect sizes from the same study, which enables the inclusion of all relevant effect sizes and thereby enhances statistical power (Assink & Wibbelink, 2016).

To conclude, our results suggest that ACT is an effective intervention for reducing psychopathology and increasing ACT related processes, well-being and coping in TAY, immediately after the intervention and at follow-up. Specifically, ACT is as effective as CBT and other treatments (e.g., REBT) and more effective than TAU and waitlist. In line with the transdiagnostic nature of ACT, the intervention works well for almost all of our (sub) outcomes (i.e., only for externalizing symptoms more research is needed). Results show that ACT is both effective when carried out in a group format as well as an individual format. Moreover, although more research is warranted, it seems that alternative forms of ACT (e.g., online and brief ACT interventions, as well as ACT interventions targeting some of the six psychological flexibility processes) may also yield positive effects for TAY. Finally, ACT is effective across different ages and in clinical, sub-clinical and non-clinical populations. Overall, these results support the use of ACT (including its alternative forms) as a transdiagnostic prevention and/or intervention method in TAY with various types and severity levels of problems.

## Search Strategy

### PsycINFO

(acceptance and* commitment.ab,id,ti. or acceptance & commitment.ab,id,ti. or acceptance commitment.ab,id,ti. or "acceptance and commitment therapy"/) and ((trial* or nonrandom* or non random* or non-random* or control* or random* or experiment* or quasi* or group* or single-blind* or single blind or waiting list or wait list or waitlist or treatment as usual or TAU or care as usual or CAU or RCT or allocat* or assign* or compar* or distribut*).ab,id,ti. or experimental design/).

## Pubmed

((“acceptance and commitment”[Title/Abstract]) OR (“acceptance commitment” [Title/Abstract]) OR (“acceptance & commitment” [Title/Abstract]) OR (acceptance and commitment therapy [MeSH Terms])) AND ((trial*[Title/Abstract]) OR (nonrandom* [Title/Abstract]) OR (“non random*” [Title/Abstract]) OR (non-random*[Title/Abstract]) OR (control*[Title/Abstract]) OR (random*[Title/Abstract]) OR (experiment*[Title/Abstract]) OR (quasi*[Title/Abstract]) OR (group*[Title/Abstract]) OR (single-blind*[Title/Abstract]) OR (“single blind” [Title/Abstract]) OR (“waiting list” [Title/Abstract]) OR (“wait list” [Title/Abstract]) OR (waitlist[Title/Abstract]) OR (“treatment as usual” [Title/Abstract]) OR (TAU[Title/Abstract]) OR (“care as usual” [Title/Abstract]) OR (CAU[Title/Abstract]) OR (RCT[Title/Abstract]) OR (allocate*[Title/Abstract]) OR (assign*[Title/Abstract]) OR (compar*[Title/Abstract]) OR (distribut*[Title/Abstract]) OR (clinical trial [MeSH Terms]) OR (experimental design [MeSH Terms])).

## Web of Science

(TS = ("acceptance and commitment" OR “acceptance commitment” OR “acceptance & commitment”)) AND (TS = (trial* OR nonrandom* OR “non random*” OR non-random* OR control* OR random* OR experiment* OR quasi* OR group* OR single-blind* OR “single blind” OR “waiting list” OR “wait list” OR waitlist OR “treatment as usual” OR TAU OR “care as usual” OR CAU or RTC or assign* or allocat* or compar* or distribut*)).

## Scopus

TITLE-ABS-KEY ({acceptance and commitment}f OR {acceptance commitment} OR {acceptance & commitment}) AND TITLE-ABS-KEY (trial* OR nonrandom* OR {non random*} OR {non-random*} OR control* OR random* OR experiment* OR quasi* OR group* OR {single-blind*} OR {single blind} OR {waiting list} OR {wait list} OR waitlist OR {treatment as usual} OR TAU OR {care as usual} OR CAU OR RTC OR assign* OR allocate* OR compar* or distribut*).

## Outcome Clustering

See Tables

**Table 5:** Sub Outcomes of Psychopathology

Internalizing problems	Externalizing symptoms	Other
Academic concern/distress	Aggressiveness	Alcohol use
Affective problems	Anger	Distress and functional impairment
Anxiety	Attention deficit hyperactivity disorder symptoms	Eating concern
Attachment anxiety	Conduct problems	Emotional and behavioural functioning
Burnout	Hostility	Emotional breakdown syndrome
Concern about negative social outcomes	Oppositional defiant problems	General mental health
Dejection		Hoarding
Depression		Insomnia
Emotional symptoms		Mental health symptoms
External shame		Pain during intercourse
Fear and avoidance of social situations		Pain during tampon test
Fear during tampon test		Positive mental health
Fear of negative evaluation		Psychiatric problems
Fear of pregnancy		Psychological health
Hair pulling severity		Psychopathology
Internalizing symptoms		Psychotic experiences
Likelihood of negative social experiences		Somatic problems
Negative affect		
Number of hairs pulled		
Number of urges to pull		
Obsessive compulsive disorder		
Positive affect		
Pregnancy related anxiety		
Psychological distress		
School stress		
Social anxiety		
Social appearance anxiety		
Stress		
Symptoms of eating disorders		
Test Anxiety		
Weight Concerns		
Worry		

5,

**Table 6:** Sub Outcomes of ACT related processes

Psychological flexibility	Self-compassion
Acceptance	Self-compassion
Anxiety-related cognitive fusion	
Body Image Avoidance	
Cognitive fusion	
Commited Action	
Defusion from negative internal experiences	
Experiential avoidance	
Mindful awareness	
Mindfulness	
Pain acceptance	
Physical activity experiential acceptance	
Psychological flexibility	
Psychological flexibility with body image	
Psychological inflexibility	
Valued living	
Valued self-care	
Values positive motivation	
Values success	

6,

**Table 7:** Sub Outcomes of Well-being

General well-being	Social well-being
Body dissatisfaction	Affection to others/own
Emotional well-being	Control to others/to own
Excitement	Empathy
General psychological well-being	Inclusion to others/to own
Happiness	Interpersonal problems
Health-related behaviour	Peer relation problems
Life satisfaction	Prosocial behaviour
Psychological well-being	Social relations
Quality of life	Social adjustment
Satisfaction with health related behaviour	Social functioning
Satisfaction with quality of life	Social well-being
Self-esteem	
Sense of coherence	
Well-being	
Welness	

7 and

**Table 8:** Sub Outcomes of Coping

Emotional coping	Cognitive coping
Difficulties in emotion regulation	(Im)mature defense style
Emotion regulation	Mental time travel
Emotional adjustment	Negative appraisals of intrusive thoughs
Interpersonal problem-solving skills	Neurotic defense style
	Perceptions related to uncertainty
	Perfectionism
	Repetitive negative thinking
	Rumination

8

## Appendix C

### Results of the Sensitivity Analysis Without Outliers

See Table

**Table 9:** Meta-Regression Results: All Significant Predictors in One Model

Predictors	*B*_*1*_ (95% CI)	*t*_*1*_	*p*
*Type of psychopathology (other*^*1*^*)*			
Externalizing symptoms	–0.29 (–0.57;0.02)	–2.08	.039
Internalizing symptoms	–0.22 (–0.38; –0.07)	–2.80	.006
*Guidance (guided*^*1*^*)*			
Unguided	0.07 (–0.46;0.60)	0.27	.786
*Delivery format (offline*^*1*^*)*			
Online	–0.19 (–0.62;0.24)	–0.88	.378
Blended	–0.82 (–1.43; –0.20)	–2.63	.009
*Therapy format (group*^*1*^*)*			
Individual	–0.20 (–0.63;0.24)	–0.88	.379
*Control group (waitlist*^*1*^*)*			
TAU	–0.04 (–0.62;0.53)	–0.15	.883
CBT	–0.81 (–1.14; –0.47)	–4.74	< .001
Other treatment	–0.47 (–0.84; –0.10)	–2.49	.014
Other	–0.52 (–1.08;0.03)	–1.85	.065
*Type of target group (nonclinical*^*1*^*)*			
Subclinical	0.29 (–0.07;0.66)	1.58	.115
Clinical	0.19 (–0.38;0.75)	0.65	.520
Mixed	0.03 (–0.90;0.97)	0.07	.944
*Gender*	0.01 (0.00;0.02)	2.55	.012

CBT, cognitive behavioural therapy; CI, confidence interval; B_1_, estimated regression coefficient; p, *p*-value of *t*-test; TAU, treatment as usual; *t*-values, difference in mean g with zero

^1^reference category

9
